# Exploring Changes in Musical Behaviors of Caregivers and Children in Social Distancing During the COVID-19 Outbreak

**DOI:** 10.3389/fpsyg.2021.633499

**Published:** 2021-03-24

**Authors:** Fabiana Silva Ribeiro, Thenille Braun Janzen, Luisiana Passarini, Patrícia Vanzella

**Affiliations:** ^1^Department of Social Sciences, University of Luxembourg, Esch-Sur-Alzette, Luxembourg; ^2^Center for Mathematics, Computing and Cognition, Universidade Federal do ABC, Santo André, Brazil; ^3^Interdisciplinary Unit for Applied Neuroscience, Universidade Federal do ABC, Santo André, Brazil

**Keywords:** COVID-19, caregiver, children, musical behavior, social distance

## Abstract

The coronavirus disease 2019 (COVID-19) pandemic has had profound effects on all aspects of society. Families were among those directly impacted by the first measures imposed by health authorities worldwide to contain the spread of the Sars-CoV-2 virus, where social distancing and mandatory quarantine were the main approaches implemented. Notably, little is yet known about how social distancing during COVID-19 has altered families' daily routines, particularly regarding music-related behaviors. The aim of this study was 2-fold: (i) to explore changes in families' daily routine and caregivers' levels of well-being and stress during the COVID-19 outbreak in Brazil and (ii) to investigate whether musical behaviors of caregivers and the children under their care (aged from 3 to 6 years old) changed during social distancing. One hundred and eighty-eight caregivers residing in Brazil participated in an online cross-sectional study conducted between July and August 2020. Our findings suggest significant changes in families' dynamics during the COVID-19 outbreak, with parents (especially mothers) spending more time on childcare and a substantial decrease in caregiver's well-being. Regarding music-related behaviors, our results revealed considerable changes in caregivers' and children's musical activities at home during social distancing, including an increase in child-only musical behaviors and shared caregiver-child activities. Moreover, sociodemographic factors such as caregiver background and well-being as well as the child's disability status significantly influenced musical engagement at home during social distancing. This study captured some features of the home musical environment of middle-class families in Brazil in the first stages of social distancing restrictions during the pandemic and caregivers' role in providing an environment where musical experiences are nurtured. Further research is needed to better understand aspects such as the long-term impact of the changes of musical behaviors at home on musical parenting and families' well-being.

## Introduction

The coronavirus disease 2019 (COVID-19) caused by the novel SARS-CoV-2 virus quickly became a global public health emergency with profound effects on all aspects of society, including psychological and social impacts (Galea et al., 2020; Holmes et al., 2020; Serafini et al., 2020; Torales et al., 2020). Families were among those directly and primarily affected by the first measures imposed by health authorities to contain the spread of COVID-19, with schools and childcare centers closures as well as social distancing recommendations rapidly disrupting the daily routine of parents and children worldwide. According to a report published by the Organization for Economic Cooperation and Development (OECD), by the end of June 2020, school closures ranged between 7 and 19 weeks across the 46 OECD and partner countries covered in the report (OECD, 2020; Schleicher, 2020). In Brazil, where this study was conducted, local authorities ordered school closures as of early March 2020, with most schools remaining closed nationwide at the time of writing this paper in October 2020 (UNESCO, 2020).

Growing evidence indicates that parents and caregivers have faced significant COVID-19-related stressors, including job loss, income loss, and caregiving burden, with a substantial increment in parent-reported daily negative mood since the start of the COVID-19 outbreak (Gassman-Pines et al., 2020; Russell et al., 2020). Recent reports suggest that the added responsibility for managing their child's schooling has significantly increased parental levels of stress, worry, social isolation, and domestic conflict, with parents of younger children reporting notably more problems relating to daily life functioning than parents of older children (Thorell et al., 2020). There is also strong evidence that parental levels of stress and well-being have a cascading effect on children and other family members, markedly affecting the household dynamics and parent-child relationships during social distancing, particularly for families undergoing financial hardships and those with single parents and children with disabilities (Brown et al., 2020; Gassman-Pines et al., 2020; King et al., 2020; Russell et al., 2020; Westrupp et al., 2020; Yoshikawa et al., 2020).

Prolonged school closures, home confinement, and family hardships during COVID-19 also affect children's physical and mental health (Fegert et al., 2020; Liu et al., 2020; Wang et al., 2020), with children with pre-existing cognitive or mental health conditions having increased difficulties during the pandemic (Thorell et al., 2020; Westrupp et al., 2020). For instance, a study conducted in Italy and Spain showed that 85.7% of parents identified noticeable emotional changes in their child's mental health, including difficulty concentrating, boredom, and feelings of loneliness (Orgilés et al., 2020). A study by Patrick et al. (2020) also revealed substantial worsening of parents and children's mental health since the beginning of social restriction measures in the USA, suggesting a potentially global issue.

Notably, little is yet known about how social distancing during COVID-19 has altered families' daily routines, particularly caregivers' and children's music-related behaviors at home, such as listening to music, singing, dancing, everyday practices, and joint-musical activities. Preliminary data suggest that there has been significant growth in the number of streaming of children's music in the USA during the period of quarantine and social distancing (Dinges, 2020). In Brazil, reports from music streaming platforms also revealed an increase in consumer's interest in relaxing and instrumental music, as well as in music and other audio content (e.g., podcasts) specifically developed for children after social distancing measures were recommended (Dezzer, 2020; Sobota, 2020). Findings such as these raise important questions about whether musical behaviors at home have changed during social distancing and whether caregivers might be intentionally using music as a tool to help cope with different challenges during the COVID-19 outbreak.

Music and musical experiences are ubiquitously prevalent in young children's lives (Custodero et al., 2003; Young, 2003, 2016; Gluschankof, 2005; Lamont, 2008; Ilari and Young, 2016). In this study, we focused specifically on the musical experiences of children between 3 and 6 years old—a transition phase that spans from toddlerhood to the age at which children usually start formal schooling (Young, 2003). Children at this age become increasingly more independent and competent, while still very connected and influenced by parents' beliefs and practices concerning music (Young, 2003; Ilari and Young, 2016). Everyday musical activities of 3–6-year olds take place in a wide range of places and situations (Lamont, 2008; Lum, 2008; Ilari and Gluschankof, 2009; Young and Ilari, 2019; Young and Wu, 2019). Nevertheless, most of the musical experiences of children under the age of 6 years consist primarily of informal musical interactions in the home environment, involving a broad range of spontaneous responses to music, such as singing along to their favorite tunes and playing musical games with caregivers or siblings (Young, 2003, 2012; Flohr, 2005; Lamont, 2008; Addessi, 2009; Barrett, 2009; Ilari and Gluschankof, 2009).

Musical parenting plays a central role in childhood musical experiences. A robust body of literature demonstrates that caregivers of children under the age of 6 years tend to interact musically with their children (Custodero and Johnson-Green, 2003; Young, 2003; Ilari, 2005; Addessi, 2009; Barrett, 2009; de Vries, 2009; Mehr, 2014; Pitt and Hargreaves, 2017), although the frequency of joint musical interactions seems to progressively diminish as the child reaches toddlerhood (Custodero et al., 2003; Kreutz and Feldhaus, 2020). Caregiver-child musical activities in the preschool years involve, for instance, joint and supported singing, improvising songs to accompany everyday routines, playing musical toys and instruments with the child, dancing, and listening to recorded music (Custodero and Johnson-Green, 2003; Addessi, 2009; Barrett, 2009; Trehub et al., 2015; Corbeil et al., 2016; Politimou et al., 2018; Cirelli et al., 2019). Studies have shown that these musical activities have important effects on family dynamics and relationships (Kreutz and Feldhaus, 2020) and provide a meaningful context to foster caregiver-child engagement across families of young children with and without developmental disabilities (Thompson, 2014; Thompson et al., 2019; Lense and Camarata, 2020; Steinberg et al., 2020). Interestingly, Saarikallio (2009) reported that caregivers of children between the ages of 3 and 8 years often also use music for mood regulation purposes.

Given the importance of musical activities within family environments and the role of parents/caregivers in early childhood musical experiences, particularly of children under the age of 6 years, we were interested in whether changes in families' dynamics imposed by school closures, social distancing, and mandates of quarantine due to the COVID-19 pandemic affected home music-related behaviors of caregivers and children in Brazil. It is well-established that musical parenting/caregiving of young children is influenced by several factors, including caregivers' previous musical experiences and socioeconomic status, family setting, culture, ethnicity, religion, lifestyle, technologies, among others (Custodero and Johnson-Green, 2003; Ilari, 2005, 2017; Hartas, 2011; Mehr, 2014). Earlier research on musical parenting/caregiving of infants and young children in Brazil revealed significant similarities between musical beliefs and practices of urban middle-class Brazilian caregivers with those of other Western countries (Ilari et al., 2011; Ilari and Young, 2016, p. 104). However, some particularities need to be considered, such as the role of extended family in the lives (including musical lives) of young children (Ilari et al., 2011).

Extended family (e.g., grandparents, aunts, older siblings), and in some cases, nannies, maids, or neighbors, are known to play an essential role in child-rearing in Brazil (Carlo et al., 2007; Cardoso and Brito, 2014; Petrucci et al., 2015; Deus and Dias, 2016; Tudge et al., 2018). One of the factors that may help to understand this dynamic is the access (or lack thereof) of Brazilian children to formal schooling in the early years (Ilari, 2007). Early childhood education and care (ECEC) in Brazil is divided into two main stages: 0–3-year olds receive ECEC in nurseries or daycare centers, and 4–6-year olds are compulsorily enrolled in preschools. According to recent estimates of the Brazilian Institute of Geography and Statistics (IBGE), 14.4% of 1-year olds and 55.4% of children between 2 and 3 years old were enrolled in formal early childhood education in Brazil in 2019 (IBGE, 2020). Among children between the ages of 4 and 5 years, it is estimated that 92.9% were enrolled in primary education in that year (IBGE, 2020). Among the reasons for the low enrolment of children below the age of 4 years old is the lack of access to publicly funded daycares/schools and after-school programs, and the parental belief that young children are better assisted at home under the care of the parents (especially the mother) or extended family (IBGE, 2020). Thus, considering the role of extended family in child-rearing and the key part they play in the everyday musical lives of preschool children (Ilari et al., 2011), this study addressed the broad category of caregivers instead of focusing solely on the parents.

Therefore, the present research aimed to better understand whether social distancing measures imposed by the COVID-19 outbreak have impacted caregivers' daily routine and their levels of well-being and stress and whether there were changes in music-related behaviors of both caregivers and children aged 3–6 years.

## Materials and Methods

### Study Design and Ethical Statement

We conducted a cross-sectional web-based study through a convenience sampling and snowball strategy, a form of nonprobabilistic selection. The study was approved by the Ethics Committee of the Universidade Federal do ABC (process: 4.133.639). The data was collected between 11 July and 11 August 2020, which corresponds to ~4–5 months after social distancing/school closure measures were implemented in Brazil. Surveys were completed through an anonymous online questionnaire available in a free software Google Forms® that circulated *via* social media. We obtained electronic informed consent from all participants, and no financial compensation was given for participation.

### Participants

The study sample consisted of caregivers of children between 3 and 6 years of age residing in Brazil. A total of 188 participants completed the survey and were included in the analysis, after excluding duplicate survey entries (*n* = 24), those that did not consent to participate (*n* = 1), and incomplete surveys (*n* = 24). Respondents were 172 parents (91.50%), eight grandparents (4.25%), seven uncles/aunts (3.72%), and one sister (0.53%). From the total cohort of caregivers, 166 were females (88.77%) aged 20–60 years (M = 36.82, SD = 6.71) and 22 were males (11.22%) aged 23–50 years (*M* = 35.64, SD = 55.56). Concerning participants' education, 52.13% had a postgraduate degree, 27.66% had completed an undergraduate degree, and 20.21% had secondary education. Participants were residents of the following demographic regions in Brazil: Southeast (77%), South (8%), Northeast (9%), North (3%), and Central-West (3%). Regarding family monthly income, 6% earned less than one minimum wage, 23% earned between one and three minimum wages, 22% between three and six minimum wages, 21% between six and 10 minimum wages, and 28% earned more than 10 minimum wages (one minimum monthly wage equals 1,045.00 BRL/~197.68 USD).

The average number of children between 3 and 6 years old per household was 1.20 (SD = 0.45; ranged from one to four children). Specifically, 81% of caregivers indicated having a single child aged 3–6 years under their care, while 17% reported caring for two children and 2% indicated caring for three and four children, totaling 225 children. Regarding children's characteristics, 107 were boys and 118 girls, with an average age of 4.32 years (SD = 1.30). Concerning the number of children attending educational or daycare institutions during the school year of 2020, 92% were formally enrolled in preschool or daycare centers while 8% were not attending educational or daycare institutions. Furthermore, 30.48% of the children (*n* = 57) within our sample presented a developmental disability, autism spectrum disorders (*n* = 30) being the most reported condition. The demographic characteristics of the study sample are available as Supplementary Table 1.

### Material

The online survey included a total of 72 questions. An *ad hoc* questionnaire consisting of 20 items evaluated caregivers' and children's sociodemographic profiles as well as the effects of social distancing on the daily habits and behaviors of the caregiver and the children (daily routine, physical and leisure activities, concentration capacity, sleep quality).

To gather information on caregivers' well-being and stress levels, the Perceived Stress Scale (PSS-10) questionnaire (Siqueira Reis et al., 2010) and the Five Well-Being Index (WHO-5) (Topp et al., 2015) were administered. The Stress Perception Scale is a self-report instrument consisting of 10 items measuring stress perception in the last 7 days on a 5-point Likert scale, where scores above 27 indicate high perceived stress levels. The Five Well-Being Index (WHO-5) is a five-item questionnaire that assesses the degree of well-being in the last week on a 5-point Likert scale, where scores below 13 indicate low levels of well-being (de Souza and Hidalgo, 2012). Cronbach alpha coefficients presented satisfactory reliability for both scales (PSS-10 = 0.84 and WHO-5 = 0.91).

To assess caregivers' and children's musical behaviors at home during social distancing, participants responded to questions adapted from the Music@Home-Preschool questionnaire (Politimou et al., 2018) and the Music Engagement Questionnaire (Vanstone et al., 2016) measuring the frequency of musical behaviors of the caregivers, the child/children (e.g., singing, listening to music, dancing, playing with musical instruments/toys), and caregiver-child musical activities. We were interested in changes in the frequency caregivers and their children engaged in different musical behaviors at home during social distancing compared with a typical day before social distancing recommendations. Specifically, seven items of the questionnaire assessed caregivers' musical behaviors, 23 items examined changes in child and caregiver-child musical activities, and five items related to children's engagement with non-musical activities. Items were rated on a 5-point Likert scale ranging from much less (1) to much more (5). Respondents also had the option to indicate if an item had never been part of the caregiver's or the children's usual musical behaviors. The translated questionnaire items and response frequencies are available as Supplementary Tables 2–5.

### Data Analysis

In the initial set of analyses, we explored whether there were changes in caregivers/children's daily behaviors and the caregivers' levels of well-being and stress during COVID-19 social distancing measures. Since we detected a high number of children with developmental disorders within our sample, we performed separate exploratory analyses using Chi-square tests (χ^2^) to investigate whether their interaction with the caregivers changed during social distancing measures. For this, we included the child's developmental disability status as independent variable and as dependent variables, the sociodemographic variables (i.e., such as age group, education, professional sector, marital status). Moreover, we also explored whether caregivers' well-being and stress levels were influenced by sociodemographic factors, by COVID-19 related questions (i.e., caregiver or a close family member with diagnosis of COVID-19, respondent or close family member considered in the risk group for COVID-19, changes in family income due to social distancing), by the presence of children with developmental disability in the household, and by daily habits and behaviors of the caregiver and the children (items displayed in the Supplementary Table 2).

In the second set of analyses, we investigated whether significant changes occurred in caregivers' and children's musical behaviors during social distancing due to COVID-19. In this context, we computed the frequencies of responses for the questionnaire's specific items (Supplementary Tables 3, 4).

Furthermore, we performed Chi-square tests (χ^2^) to investigate how the following independent variables could influence musical behaviors of children/caregivers during social distancing measures: caregivers' experience and current involvement with artistic activities (visual arts, dance, theater, music), caregiver's level of education, caregivers' well-being and stress, and the child's disability status. For this analysis, participant's responses were grouped into four frequency categories (never/less/no change/more). It is important to note that the items in our questionnaires were analyzed separately as we did not incorporate all the items contained in the Music@Home-Preschool questionnaire (Politimou et al., 2018) and the Music Engagement Questionnaire (Vanstone et al., 2016), as well as the response options on the scales were modified in order to reflect the change in behaviors during COVID-19. Moreover, our sample size and the number of items surveyed did not allow us to perform a factorial analysis to cluster or reduce the amount of statistical analysis performed. Therefore, we did not assume a total score or that some items would be assessing the same underlying constructs.

To prevent type I error, each individual analysis carried out with the χ^2^ test was adjusted with Bonferroni's correction using *p* = 0.05 as the main level of significance divided by the multiplication of the number of dependent variables vs. independent variables in each analysis. Furthermore, we measured effect size for the χ^2^ test using Cramer's V. Descriptive statistics were generated for all variables, but only statistically significant findings after Bonferroni adjustments are reported. Statistical analysis was carried out using Stata 16 (Stata, College Station, Texas).

## Results

### Changes in Daily Behaviors and Caregivers' Reported Levels of Well-Being and Stress

As detailed in Table 1, before the COVID-19 outbreak, it is possible to observe that different caregivers were involved in child-rearing responsibilities, including extended families and external helpers. However, with the imposition of COVID-19 social distancing measures, survey responses suggest a reduction of care from non-parents and an increase in the number of parents who became the primary carers during the pandemic.

**Table 1:** Main caregiver before and during the COVID-19 outbreak (*n* = 188).

**Main caregiver**	**Before COVID-19**	**During COVID-19**
Parents (mother and/or father)	113 (60.11%)	131 (69.68%)
Parents and extended family	45 (23.93%)	42 (22.34%)
Extended family	21 (11.17%)	10 (05.32%)
Parents and external	03 (01.60%)	04 (02.13%)
External	06 (03.19%)	01 (00.53%)

*Frequency (percentage). External: nanny, maid, or neighbor*.

Results also revealed that 81.38% of caregivers reported spending more time with their child(ren) during social distancing measures and 60% of caregivers reported that staying at home with their child(ren) had become more difficult with time (Supplementary Table 2). Further analysis showed that caregivers of children with developmental disabilities reported more frequently increased difficulty in staying at home with their child during social distancing (71.93%) compared with caregivers of typically developing children (54.62%), χ^2^ = 10.29, *p* = 0.006, Cramer's *V* = 0.19.

Regarding caregiver well-being and stress levels, respondents' average score on the well-being scale was 12.80 (*SD* = 5.19), with 49.47% (*n* = 93) revealing low levels of well-being. Caregivers' average score on the perceived stress scale was 22.05 (*SD* = 5.19), with 23.94% (*n* = 45) showing severe stress levels during social distancing. However, our analyses did not reveal statistically significant outcomes when exploring independent variables such as stress levels (severe and no stress levels) and well-being (low and typical levels) vs. dependent variables, namely demographic factors and COVID-19-related items.

### Changes in Musical Behaviors of Caregivers and Their Children

**Figure 1:**
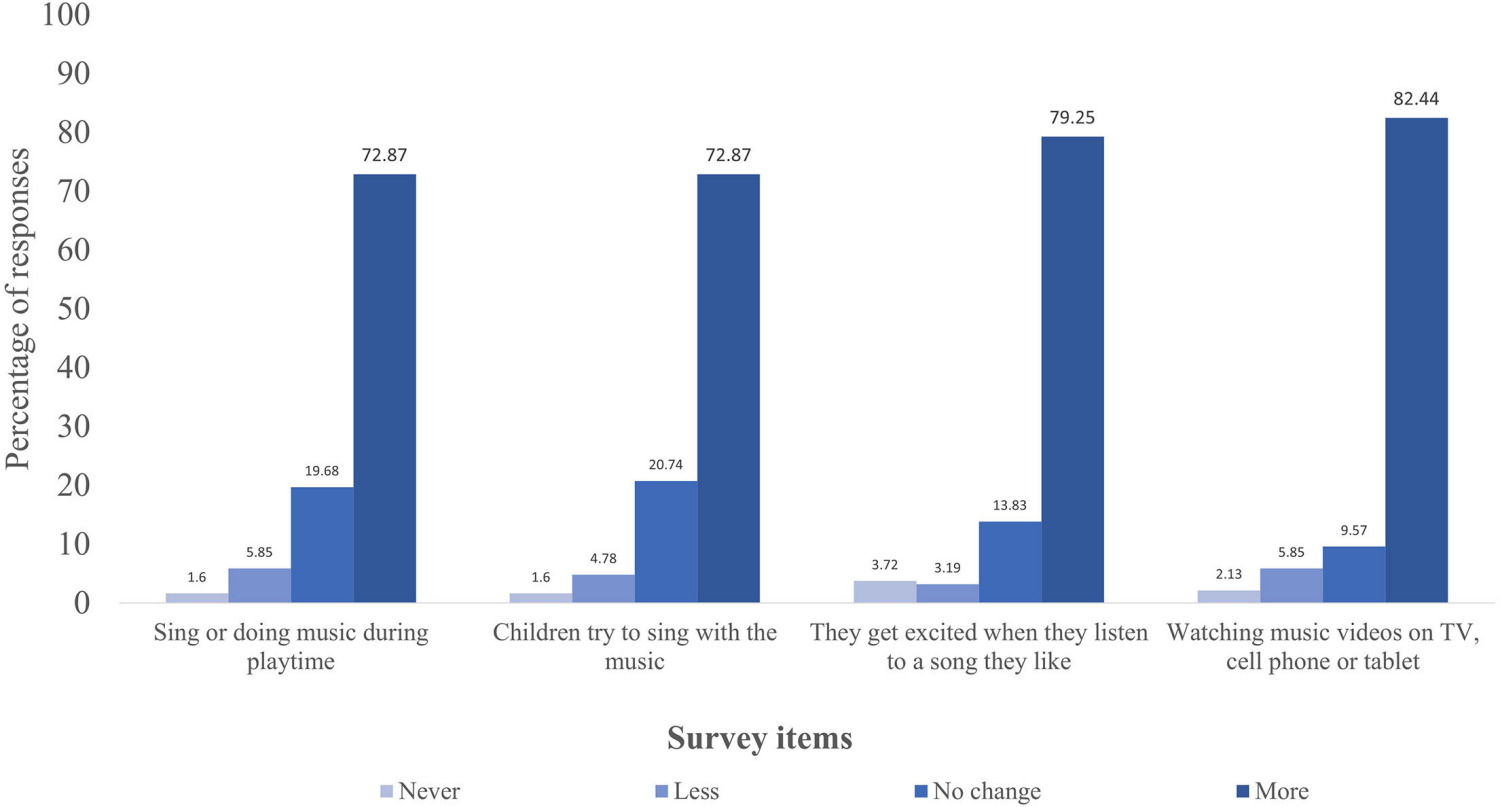
Percentage of responses regarding changes in children's musical behaviors during social distancing due to COVID-19 measures.

**Figure 2:**
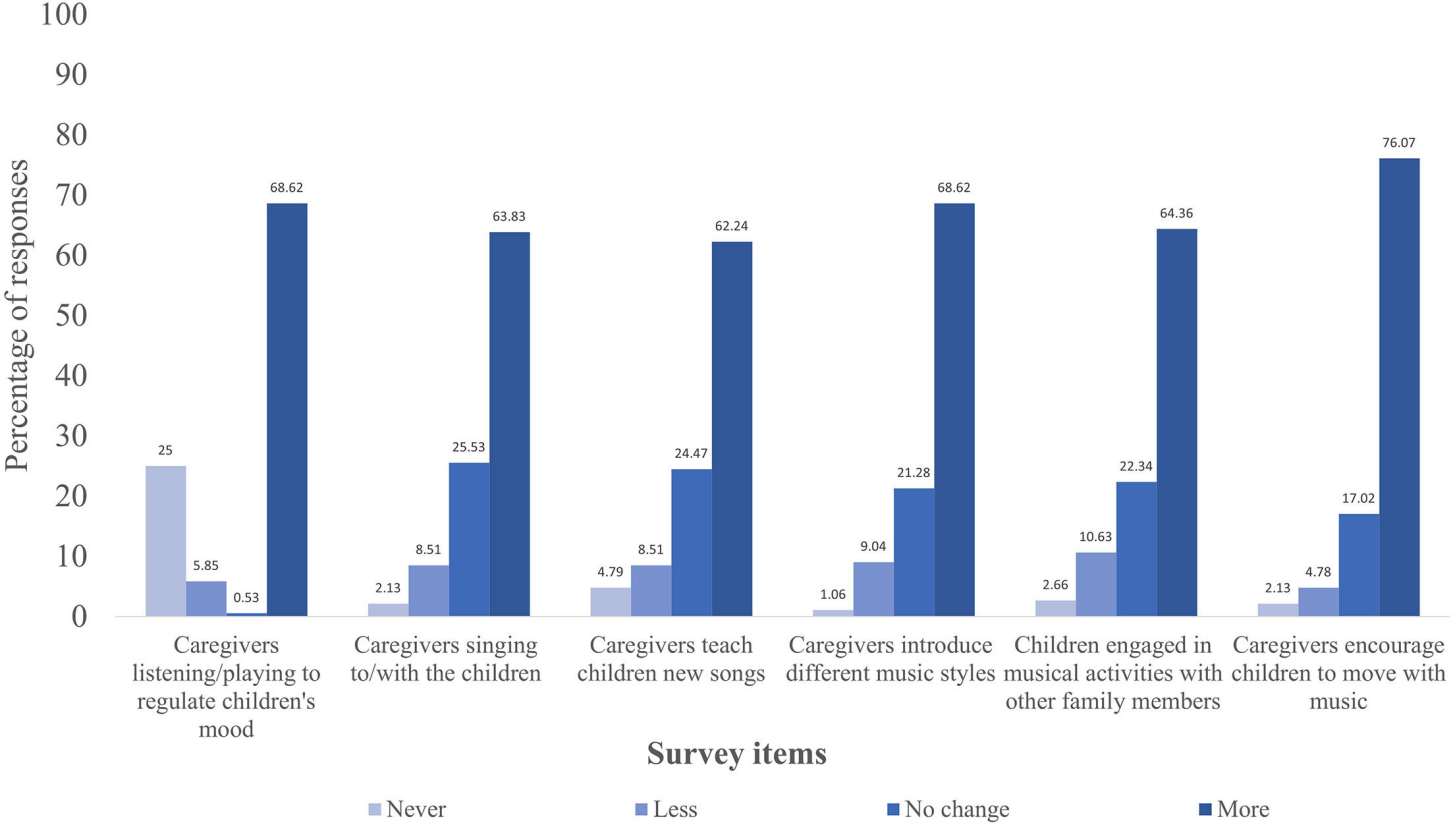
Percentage of responses regarding changes in shared caregiver-child musical experiences during social distancing due to COVID-19 measures.

For a simplified graphical depiction of the results, we grouped participants' responses into four frequency categories (never/less/no change/more) and only display the survey items with the highest percentage of change related to caregiver-reported children' activities (Figure 1) and child-caregiver musical behaviors (Figure 2). Specifically, Figure 1 displays items with responses above 70% and Figure 2 depicts item responses above 60%. Response frequencies for each surveyed item are available as supplementary materials (Supplementary Tables 3, 4).

Frequency measures suggest relevant changes in musical behaviors for both caregivers and children during social distancing. It was found that 57.44% of caregivers reported listening to music more frequently during social distancing in comparison with a typical day before social restrictions. Results also suggest that most respondents intentionally used music for mood regulation, as ~60% of caregivers indicated that they listened to music to relax or feel excited during social distancing (Supplementary Table 3).

Survey responses also indicated considerable changes in children's musical behaviors, with caregivers revealing an increase in virtually all items surveyed (Supplementary Table 4). For instance, caregivers reported that, during social distancing, children engaged more frequently in activities such as dancing or moving to music (67.55%), listening to recorded music (67.02%), engaging in music-related activities with another person (64.36%), listening to music to calm down (65.95%), singing by themselves (62.77%), and creating their own music (65.43%) compared with a typical day before social restrictions (Supplementary Table 4; Figure 1). Respondents also indicated an increase in non-musical indoor activities during social distancing compared with before restrictions, including playing games (81.91%) and drawing/painting (77.13%) and a general decrease in outdoor activities (60.63%) and physical activities (65.42%) (Supplementary Table 5).

Additionally, results suggest an increase in shared caregiver-child musical experiences during social distancing compared with before the restrictions. As depicted in Figure 2, caregivers reported that their child/children engaged more often in musical activities with other family members during social distancing (64.36%) and that they listened to music (57.44%) and sang to/with the child/children (63.83%) more frequently than before social restrictions. Most caregivers also answered that they played recorded music or played a musical instrument to regulate their child's mood more often during social distancing (68.62%) compared with before the COVID-19 outbreak.

To explore the association between different caregiver psychosocial factors and musical behaviors during social distancing, we first assessed the impact of caregivers' experience and current involvement with artistic activities (visual arts, dance, theater, music). No significant statistical effects were observed when the independent variables were caregivers with and without previous artistic experience. Nevertheless, the analysis suggested that caregivers who were currently involved in an artistic activity reported that they intentionally motivated their children to dance or move with music (87.84%) more often than caregivers not engaged in artistic practice (73.85%), χ^2^ = 6.05, *p* = 0.04, Cramer's *V* = 0.17.

Results also revealed a significant effect of caregiver's level of education on musical behaviors during social distancing. We observed that 73.68% of caregivers with a postgraduate degree indicated singing more often to/with their children during social distancing compared with caregivers with secondary (64.86%) and undergraduate education (50%), χ^2^ = 11.14, *p* = 0.02, Cramer's *V* = 0.18. Similarly, 77.32% of caregivers with a postgraduate degree tended to expose their children to different music styles more often than caregivers with an undergraduate degree (53.85%), χ^2^ = 10.99, *p* = 0.03, Cramer's *V* = 0.17.

Caregiver well-being and stress also seem to influence musical behaviors at home during COVID-19. The statistical analysis indicated that higher levels of reported well-being were significantly associated with more engagement with music-related activities during social distancing. Specifically, 80.77% of caregivers with higher levels of well-being reported spending more time listening to or playing music to socially connect with other adults in the family during social distancing compared with 47.73% of caregivers with lower levels of well-being, χ^2^ = 11.73, *p* = 0.001, Cramer's *V* = 0.35. Furthermore, 55.07% of caregivers presenting higher levels of well-being indicated an increased frequency of behaviors such as singing and playing musical instruments alone during social distancing compared with 37.31% of caregivers with lower well-being levels χ^2^ = 8.03, *p* = 0.02, Cramer's *V* = 0.24. However, the analysis examining the association between caregiver stress levels on their musical behaviors revealed no significant interactions between reported stress and any of the items assessed.

Concerning the influence of caregiver's well-being on children's musical behaviors, we found that caregivers with higher levels of well-being sang more often with/to their children (72.04%) than those with lower levels of well-being (58.24%), χ^2^ = 8.44, *p* = 0.01, Cramer's *V* = 0.21, as well as taught more new songs to their children during social distancing (74.73%) than caregivers with lower levels of well-being (55.68%) χ^2^ = 7.23, *p* = 0.03, Cramer's *V* = 0.20. When we analyzed caregiver stress levels' influence on changes in children's musical behaviors during COVID-19, we found no significant interactions.

Finally, the analysis exploring musical behaviors among caregivers of children with developmental disabilities showed that 77.78% of children with developmental delays were more involved in musical activities with another person (dancing, singing, listening to music, and playing music) during social distancing compared with 60.94% of typically developing children, χ^2^ = 7.06, *p* = 0.03, Cramer's *V* = 0.19. Survey responses also suggest that children with developmental delays (20.41%) were less engaged in creative musical activities (e.g., making up their songs) during social distancing than children with typical development (3.33%), χ^2^ = 11.89, *p* = 0.003, Cramer's *V* = 0.28.

## Discussion

In this cross-sectional study, we explored whether social distancing during the COVID-19 outbreak in Brazil impacted caregivers' daily routine as well as their levels of well-being and stress, and whether the musical behaviors of caregivers and their children (aged 3–6 years old) at home changed during social distancing. Our findings suggest important changes in families' dynamics during the COVID-19 outbreak, with parents (especially mothers) spending more time on childcare, and a significant decrease in caregiver's well-being. Regarding music-related behaviors, our results revealed significant changes in caregivers' and children's music activities at home during social distancing measures, with an increase in musical engagement associated with sociodemographic factors such as caregivers' background (i.e., current experience with artistic activities and educational level), caregiver well-being, and the child's disability status.

### Effects on Daily Routine and Caregivers' Levels of Well-Being and Stress

Our results indicated that, after school closures and social distancing recommendations, children in our sample were primarily cared for by their parents (especially mothers), with the vast majority of parents indicating that they were spending more time with their child during social distancing measures compared with a typical day before restrictions. This result agrees with a worldwide trend during the COVID-19 pandemic (Power, 2020; Thorell et al., 2020; Yoshikawa et al., 2020). One of the factors that may be directly related to this finding is the large societal disparities based on gender across society, with women having the main responsibility for unpaid care activities, including childcare (King et al., 2020).

In our sample, we also detected that caregivers of children with developmental disabilities reported greater difficulty staying at home with the child during social distancing than caregivers of typically developing children. It is important to highlight that caregivers of these children, in many cases, received support from specialized services before the pandemics and may have been particularly affected by the added responsibilities for managing their child's schooling and care with no specific training to attend to their children's special needs (Aishworiya and Kang, 2020). Moreover, children with developmental disorders, such as Autism Spectrum Disorder (ASD), may have additional difficulties comprehending the recent changes in their daily routine, adding to the confluence of stressors faced by families with children with additional needs (Aishworiya and Kang, 2020).

Concerning the higher demands imposed by the pandemic in caregivers' life, there is a well-established body of studies showing that conflicts between work and caregiver roles are associated with increased mental health problems (Patrick et al., 2020; Van Tilburg et al., 2020; Westrupp et al., 2020). Indeed, we observed that 49.5% of caregivers reported low levels of well-being and 23.9% reported severe stress levels, which is consistent with previous studies indicating that the COVID-19 pandemic can have significant effects on adults' mental health (Patrick et al., 2020; Russell et al., 2020). Although we do not know whether the low levels of reported well-being or stress were pre-existing conditions, these results are congruent with a study suggesting a high prevalence of mental issues in Brazilian adults during the COVID-19 pandemic (Campos et al., 2020).

Finally, it is also important to mention that caregiver stress and low levels of well-being can significantly affect children's development (Fisher et al., 2020; Yoshikawa et al., 2020). According to Fisher et al. (2020), stressful experiences in the early years of life can have an enduring influence on the development of the brain as well as children's biology in later life, consequently enhancing the risk for emotional disorders, learning difficulties, and health problems. For this reason, our outcomes highlight the importance of developing preventive measures to improve the psychological aspects of caregivers in Brazil.

### Caregivers' and Children's Musical Behaviors During Social Distancing

Our findings indicated significant changes in caregivers' and children's musical behaviors during social distancing due to the COVID-19 outbreak in Brazil. Results showed that caregivers tended to engage more frequently in music-related activities (e.g., listening to music) during social distancing compared with a typical day before social restrictions. This finding agrees with a general trend revealed by studies showing an increase in the time adults spent on musical activities during the COVID-19 pandemic (Cabedo-Mas et al., 2020; Krause et al., 2020).

Our results also indicated an overall increase in children's musical experiences at home during social distancing, including shared caregiver-child activities. Findings demonstrated a considerable increment in the frequency of active caregiver-child joint musical experiences during social distancing, such as dancing, singing, listening to music, and playing music.

It is possible that these results may reflect culture-specific social processes and parenting beliefs concerning music. A study by Kirschner and Tomasello (2010) assessing joint drumming in Brazilian and German preschool children noted that, while there were substantial similarities regarding general lifestyle, socioeconomic background, and children's access to musical media at home, Brazilian caregivers reported generally spending more time engaged in daily active music-making (singing, dancing, playing instruments) with their children than German parents. Similarly, Brazilian parents also seem to engage their children more often in active music-making than their German counterparts. Some scholars argue that Brazilian society is relatively more collectivist, placing more emphasis on cooperation (Carlo et al., 2007). Thus, it is possible that these cultural tendencies may be embedded in Brazilian's beliefs and practices concerning music parenting/caregiving (Ilari et al., 2011). Alternatively, it may be that caregivers found in these shared musical experiences an opportunity for safe time-passing (Schubert, 2009; Schäfer et al., 2013), entertainment (Trehub and Schellenberg, 1995), caregiver-child bonding (Creighton et al., 2013; Boer and Abubakar, 2014; Persico et al., 2017; Fancourt and Perkins, 2018a,b), or to promote individual and collective well-being (Saarikallio, 2009; Schäfer et al., 2013; Kreutz and Feldhaus, 2020).

Nevertheless, the increase in music behaviors at home during social distancing seemed to have been directly influenced by sociodemographic factors such as caregivers' background (current involvement with artistic activities and educational level), caregiver well-being, and the child's disability status.

Specifically, we found that caregivers involved in artistic activities (visual arts, dance, theater, music) during social restrictions and those with higher levels of education seemed to intentionally influence aspects of the musical home environment by encouraging their children to engage in motor activities to music (dancing and moving to the music), singing to/with the child, and exposing their children to a broad range of musical styles. These findings concur with previous evidence suggesting that factors such as caregivers' own engagement with music and educational level can significantly influence musical parenting/caregiving (Custodero and Johnson-Green, 2003; Ilari, 2005, 2017; Hartas, 2011; Mehr, 2014). Interestingly, our results suggested that caregivers currently engaged in various art-related activities (visual arts, dance, theater, music) were more likely to provide a home environment where musical experiences were nurtured during social distancing. This result is in line with earlier findings that parents' personal level of engagement with music, and not necessarily their formal musical training, seems to be a determining factor in how parents interact musically with their children (Politimou et al., 2018). Future research is needed to better address the role of caregivers' engagement in art-related activities (other than music) on their beliefs and practices relating to music at home.

Increased musical engagement during social distancing was also linked with caregivers' well-being. Our results indicated that caregivers who reported higher levels of well-being tended to engage in individual music-related activities (singing or listening to music) more often during social distancing than caregivers with lower reported well-being. Music engagement has long been associated with psychological well-being across the lifespan (MacDonald, 2013; Schäfer et al., 2013; Fancourt and Finn, 2019; Brancatisano et al., 2020), with research indicating that music is an effective tool for mood and emotional self-regulation through mechanisms such as mood improvement, distraction, and relaxation (Saarikallio, 2011; Baltazar and Saarikallio, 2016; Groarke and Hogan, 2016). Interestingly, it was also observed that caregivers with higher levels of well-being reported an increase in music-related social behaviors, such as listening or playing music to connect with others (family and non-family members), which suggests a purposeful use of music to socialize and feel connected during social distancing measures. It has been shown that social interactions through music can effectively reduce feelings of loneliness and provide a sense of togetherness and belonging (Labbé et al., 2007; Laukka, 2007; Miranda and Claes, 2009; Tymoszuk et al., 2020). This may explain a general trend observed across the globe regarding the use of music through online musical gatherings (e.g., live music streams and concerts) as a positive coping strategy to deal with feelings of loneliness and social/physical disconnection (Cabedo-Mas et al., 2020; MacDonald et al., 2020; Vandenberg et al., 2021).

Caregiver well-being also had a direct impact on children's musical engagement at home during social distancing. Survey results indicated that caregivers with higher levels of well-being tended to sing more with/to their children and reported teaching them new songs more frequently than caregivers with lower well-being. This result indicates that parental mental health is an important factor to determine musical engagement at home (Custodero et al., 2003), as parental depression is associated with disengaged parenting and reduced empathy toward their young children (National Research Council, 2009; Salo et al., 2020). Thus, it may be that caregivers with reported higher levels of well-being were simply paying more attention to their children due to their internal states, and for this reason, reported more playful behaviors.

Even though we observed that caregivers' well-being was associated with children's musical behaviors during social distancing, our findings indicated that caregivers' stress levels did not seem to interact with their own and their children's musical engagement at home. Our survey data demonstrated that self-reported caregiver stress indeed increased in response to the current situation, with 23.94% indicating severe levels of stress. Thus, it is possible that our assessment measures and study sample were not sufficient to capture an association between caregiver stress and changes in musical practices at home during social distancing. We must also consider a possible association between caregivers' mental health and their beliefs concerning music or potentially maladaptive uses of music for mood and emotion regulation (Thomson et al., 2014; Carlson et al., 2015), warranting further research.

It was also interesting to note that the child's disability status influenced musical engagement at home during social distancing measures. Our results suggested, for instance, that the frequency of shared musical interactions was even higher within families with children with developmental disabilities than with typically developing children. One possible explanation is that, with school closures and the interruption of specialized support services, caregivers may have felt the need to foster an enriched home environment to support their children's cognitive and social development. Considering that informal musical experiences at home are a common and natural form of caregiver-child interaction, shared musical activities may have been an intuitive and familiar way to provide a predictable, reinforcing, and emotionally modulating context for interaction (Lense and Camarata, 2020). Studies have indeed shown that families of preschool-aged children with disabilities do tend to incorporate music activities they experience in family-centered music therapy (signing, playing, and listening to music) into everyday life (Thompson, 2014; Lense et al., 2020; Steinberg et al., 2020). It may also be that caregivers of children with disabilities, like caregivers of typically developing children, used music at home to foster strong caregiver-child relationships (Thompson, 2014; Thompson et al., 2019; Lense and Camarata, 2020; Steinberg et al., 2020), safe time-passing and entertainment (Trehub and Schellenberg, 1995; Schäfer et al., 2013), or mood regulation (Saarikallio, 2009; Schäfer et al., 2013).

### Limitations

We acknowledge that the study sample's characteristics must be considered when interpreting the present results. Our sample consisted of well-educated caregivers who were able to work from home and care for their children during social distancing measures. Therefore, it is possible that the results of this study were skewed by aspects such as the fact that caregivers who spent long periods of time with the children at home during the COVID-19 outbreak might have had more opportunities to observe behaviors that were perhaps not evident before (Young, 2003; Addessi, 2009). We should also consider the influence of social desirability bias as it is well-established that parents/caregivers tend to overestimate the frequencies of musical behaviors to show good parenting skills (Bornstein et al., 2015; Ilari, 2017), and the fact that caregivers who generally use music in the home may have been more interested in participating in the study. In addition, this study was a web-based survey, and as such, access to technology is a limiting factor as only individuals with access to the web and time to respond to questionnaires were surveyed. Thus, a more controlled sample would help to better understand the impact of social distancing measures on musical behaviors across a wider array of families. Finally, due to this study's cross-sectional nature, a causal relationship between the observed changes in musical behaviors and social distancing during the COVID-19 outbreak cannot be determined.

## Conclusions and Prospective Studies

This study adds knowledge about caregivers and children's musical behaviors ~4–5 months into the COVID-19 outbreak in Brazil. Evidence of substantial changes in families' dynamics emerged. Parents (especially mothers) reported spending more time on childcare, which conceivably impacted caregivers' well-being. Results also suggested an overall increase in musical engagement at home during social distancing, with the frequency of music-related behaviors being significantly associated with sociodemographic factors such as caregivers' background and well-being and the child's disability status. This study thus captured some of the features of the home musical environment of urban middle-class families in Brazil during the first stages of social distancing restrictions during the COVID-19 outbreak and caregivers' role in providing an environment where musical experiences are nurtured.

These results shed some light on families' dynamics during unprecedented circumstances and how music seemed to have been a useful tool to help caregivers and their children to cope with stressors during the COVID-19 outbreak, thus, presenting some practical implications for music therapists, educators, and researchers. For instance, although we did not directly investigate what strategies were used for mood or emotion regulation, our results suggest that caregivers intentionally used music to help modulate their own and their children's mood and emotions during social distancing. This seems to be an intuitive strategy adopted by caregivers to help cope with the pandemics' psychological impact and minimize the negative impacts of COVID-19-related stressors on their own and their children's mental health and well-being. This is a direct line of action whereby music therapists can provide practical strategies to help caregivers manage their own and their children's mental health during the pandemics, providing guidelines on how to effectively use music as a self-regulation tool to modulate mood and emotions, and reduce irritability and restlessness. Music therapists are also key to assist caregivers of children with developmental disabilities in how to better accommodate their children's needs while specialized services remain closed and how to foster shared musical experiences at home as a tool to strengthen caregiver-child bonding and promote a sense of security and resilience.

There is an urgent need for prospective studies to assess outcomes longitudinally. For instance, further research is needed to better understand whether musical practices at home have changed throughout the months of social distancing or the long-term impact of musical behaviors at home on musical parenting and families' well-being. We are also yet to understand how musical-related activities compare with other forms of art-related (e.g., dancing, painting) or technology-related activities on children's physical and mental health and overall well-being. By looking at caregivers' and children's musical experiences at home during the COVID-19 outbreak, we hope that new inquiry areas may open to better understand musical experiences in the home during challenging times for families.

## Data Availability Statement

The raw data supporting the conclusions of this article is available as Supplementary Material, without undue reservation.

## Ethics Statement

The studies involving human participants were reviewed and approved by Ethics Committee of the Universidade Federal do ABC (Process: 4.133.639). The patients/participants provided their written informed consent to participate in this study.

## Author Contributions

FR analyzed and interpreted the data. FR and TJ drafted the initial manuscript. All the authors designed the study, contributed to the revision of the manuscript, and approved the submitted version.

## Conflict of Interest

The authors declare that the research was conducted in the absence of any commercial or financial relationships that could be construed as a potential conflict of interest.
