# Supplementary material for: Exploring Changes in Musical Behaviors of Caregivers and Children in Social Distancing During the COVID-19 Outbreak
Source: Front Psychol. 2021 Mar 24;12:633499. doi: 10.3389/fpsyg.2021.633499 (PMC8024569; doi:10.3389/fpsyg.2021.633499)
Supplement: Supplementary file 1 [file Data_Sheet_1.pdf]

Supplementary Material

Table S1. Sociodemographic information of the sample (*n* = 188).

**Variables**	***N* (%)**
**Age Group**	
18-34 years old	66 (35.11)
> 35 years old	122 (64.89)
Age (mean ± SD)	36.69 (6.58)
**Education**	
Secondary	38 (20.21)
Undergraduate	52 (27.66)
Postgraduate	98 (52.13)
**Professional Sector**	
Education	54 (28.72)
Health	37 (19.68)
Others	97 (51.60)
**Family income**	
1-6 minimum wages	96 (51.06)
> 6 minimum wages	92 (48.94)
**Marital Status**	
Single	14 (7.45)
Married/ *De facto* union	161 (85.64)
Divorced	10 (5.32)
Widow	03 (1.60)
**Diagnosis of COVID-19 (respondent or close family member)**	
No	131 (69.68)
Yes	57 (30.32)
**Respondent considered in the risk group for COVID-19**	
No	158 (84.04)
Yes	30 (5.96)
**Close family member in the risk group for COVID-19**	
No	120 (63.83)
Yes	68 (36.17)
**Compliance with social distancing measures**	
No	09 (4.79)
Yes	179 (95.21)

**Number of persons living in the same house (mean ± SD)**	3.79 (1.01) [range: 2 min - 7 max]
**Number of children aged between 0-12 years old living in the same house (mean ± SD)**	1.52 (0.72) [range: 1 min – 6 max]
**Number of children aged between 3-6 years old living in the same house (mean ± SD)**	1.20 (0.45) [range: 1 min – 4 max]
**Children with developmental disability**	
No	130 (69.52)
Yes	57 (30.48)
**Changes in family income due to social distancing**	
No change	95 (50.53)
Improved	14 (7.45)
Worsened	79 (42.02)
**Caregiver well-being score (WHO-5) (mean ± SD)**	12.80 (5.19)
**Caregiver stress score (PSS-10) (mean ± SD)**	22.05 (6.28)
**Caregiver experienced in an artistic activity**	
No	137 (72.87)
Yes	51 (27.13)
**Caregiver currently practicing an artistic activity**	
No	112 (59.57)
Yes	24 (12.77)
**Importance of music in the caregivers' households (mean ± SD)**	8.58 (1.73)

Table S2. Results of behavioral effects of social distancing during the COVID-19 pandemic.

Time spent with the child during social distancing for the main caregiver	
Much less	08 (04.26)
A little less	06 (03.19)
No change	21 (11.17)
A little more	43 (22.87)
Much more	110 (58.51)
Care of other adults living in the same house and time spent with the child during social distancing	
Much less	17 (09.04)
A little less	10 (05.32)
No change	35 (18.62)
A little more	40 (21.28)
Much more	70 (37.23)
There are no other adults in the house	16 (08.51)
Rated difficulty of staying at home with the child(ren)	
No change	65 (34.57)
Easier	10 (05.32)
More difficult	113 (60.11)
Work/study routine during social distancing	
No change	34 (18.09)
Improved	16 (08.51)
Worsened	138 (73.40)
Daily routine of the child(ren) (sleeping, waking up, and eating behaviors) during social distancing	
No change	68 (36.17)
Improved	21 (11.17)
Worsened	99 (52.66)
Family leisure activities during social distancing	
No change	14 (07.45)
Improved	08 (04.26)
Worsened	166 (88.30)
Physical activity of the caregiver during social distancing	
No change	40 (21.28)
Improved	18 (09.57)
Worsened	130 (69.15)
Physical activity of the child(ren) during social distancing	
No change	31 (16.49)
Improved	08 (04.26)
Worsened	149 (79.26)
Caregiver concentration during social distancing	
No change	57 (30.32)
Improved	19 (10.11)

Worsened	112 (59.57)
Child(ren)'s concentration during social distancing	
No change	87 (46.28)
Improved	17 (09.04)
Worsened	84 (04.68)
Quality of the caregiver's sleep during social distancing	
No change	84 (44.68)
Improved	17 (09.04)
Worsened	87 (46.28)
Quality of the child(ren)'s sleep during social distancing	
No change	132 (70.21)
Improved	56 (29.79)
Worsened	52 (27.66)

Note: Frequency (Percentage).

Table S3. Changes in caregivers' musical behaviors during social distancing. Questions focused on how often caregivers engaged in each item during social distancing compared to before the social restrictions imposed by health authorities in Brazil.

Listening to music	
I never had this habit	05 (02.66)
Much less	08 (04.26)
A little less	28 (14.89)
No more and no less	39 (20.74)
A little more	65 (34.57)
Much more	43 (22.87)
Singing or playing a musical instrument alone	
I never had this habit	52 (27.66)
Much less	20 (10.64)
A little less	13 (06.91)
No more and no less	40 (21.28)
A little more	42 (22.34)
Much more	21 (01.17)
Listening to music or playing a musical instrument with another adult of the family (partner, adult sibling, parent)	
I never had this habit	44 (23.40)
Much less	16 (08.51)
A little less	10 (05.32)
No more and no less	46 (24.47)
A little more	46 (24.47)
Much more	26 (13.83)
Listening to music or playing a musical instrument with another non-family adult (friends, community groups, online or social media events)	
I never had this habit	72 (38.30)
Much less	56 (29.79)
A little less	11 (05.85)
No more and no less	32 (17.02)
A little more	10 (05.32)
Much more	07 (03.72)
Listening to music or playing a musical instrument to change my mood or physical state (energize, relax)	
I never had this habit	55 (29.26)
Much less	09 (04.79)
A little less	11 (05.85)
No more and no less	01 (00.53)
A little more	57 (30.32)
Much more	55 (29.26)
Listening to music or playing a musical instrument to socially connect with other adults in the family (partner, adult sibling, parent)	

I never had this habit	92 (48.94)
Much less	17 (09.04)
A little less	16 (08.51)
No more and no less	0 (0)
A little more	38 (20.21)
Much more	25 (13.30)
Listening to music or playing a musical instrument to socially connect with other non-family adults (friends, community groups, online or social media events)	
I never had this habit	79 (42.02)
Much less	47 (25.00)
A little less	24 (12.77)
No more and no less	0 (0)
A little more	25 (13.30)
Much more	13 (06.91)

Note: Frequency (Percentage).

Table S4. Caregiver-reported changes in children and child-caregiver musical behaviors. Questions focused on how often caregivers and children engaged in each item during social isolation compared to a typical day before social restrictions imposed by health authorities in Brazil.

**Children musical behaviors**	
The child interacts or plays with musical instruments or musical toys	
Never had this habit	14 (07.45)
Much less	06 (03.19)
A little less	13 (06.91)
No more and no less	52 (27.66)
A little more	55 (29.26)
Much more	48 (25.53)
The child dances/moves to music	
Never had this habit	05 (02.66)
Much less	01 (00.53)
A little less	10 (05.32)
No more and no less	45 (23.94)
A little more	52 (27.66)
Much more	75 (39.89)
Singing and making music is a regular part of playtime	
Never had this habit	03 (01.60)
Much less	04 (02.13)
A little less	07 (03.72)
No more and no less	37 (19.68)
A little more	64 (34.04)
Much more	73 (38.83)
The child likes to listen to new songs	
Never had this habit	06 (03.19)
Much less	02 (01.06)
A little less	09 (04.79)
No more and no less	45 (23.94)
A little more	50 (26.60)
Much more	76 (40.43)
The child tries to sing along to the music that is being played	
I never had this habit	03 (01.60)
Much less	02 (01.06)
A little less	07 (03.72)
No more and no less	39 (20.74)

A little more	52 (27.66)
Much more	85 (45.21)
The child shows preferences for specific types of music	
I never had this habit	09 (04.79)
Much less	01 (00.53)
A little less	05 (02.66)
No more and no less	52 (27.66)
A little more	51 (27.13)
Much more	70 (37.23)
The child becomes emotional when (s)he hears music	
Never had this habit	12 (06.38)
Much less	06 (03.19)
A little less	08 (04.26)
No more and no less	68 (36.17)
A little more	49 (26.06)
Much more	45 (23.94)
The child stops what (s)he is doing in order to listen to music that is being played	
Never had this habit	07 (03.72)
Much less	02 (01.06)
A little less	05 (02.66)
No more and no less	53 (28.19)
A little more	55 (29.26)
Much more	66 (35.11)
The child likes to listen to music to pass the time when (s)he is bored	
Never had this habit	08 (04.26)
Much less	04 (02.13)
A little less	08 (04.26)
No more and no less	58 (30.85)
A little more	45 (23.94)
Much more	65 (34.57)
The child gets excited when the music that (s)he likes plays	
Never had this habit	07 (03.72)
Much less	02 (01.06)
A little less	04 (02.13)
No more and no less	26 (13.83)
A little more	60 (31.91)
Much more	89 (47.34)

The child creates his/her own music (e.g., by making up his/her own tunes or by changing the words to existing songs).	
Never had this habit	18 (09.57)
Much less	05 (02.66)
A little less	09 (04.79)
No more and no less	33 (17.55)
A little more	46 (24.47)
Much more	77 (40.96)
The child calms down when listens to music	
Never had this habit	08 (04.26)
Much less	01 (00.53)
A little less	09 (04.79)
No more and no less	46 (24.47)
A little more	54 (28.72)
Much more	70 (37.23)
The child sings by him/herself	
Never had this habit	05 (02.66)
Much less	06 (03.19)
A little less	10 (05.32)
No more and no less	49 (26.06)
A little more	71 (37.77)
Much more	47 (25.00)
The child listens to recorded music	
Never had this habit	02 (01.06)
Much less	13 (06.91)
A little less	47 (25.00)
No more and no less	69 (36.70)
A little more	57 (30.32)
The child watches music videos on TV/cellphone/tablet	
Never had this habit	04 (02.13)
Much less	02 (01.06)
A little less	09 (04.79)
No more and no less	18 (09.57)
A little more	69 (36.70)
Much more	86 (45.74)
The child engages in music activities with another person (e.g., dancing/singing/playing music/listening to music)	
Never had this habit	05 (02.66)

Much less	07 (03.72)
A little less	13 (06.91)
No more and no less	42 (22.34)
A little more	69 (36.70)
Much more	52 (27.66)
**Caregiver-child musical behaviors**	
I sing to/with the child	
Never had this habit	04 (02.13)
Much less	06 (03.19)
A little less	10 (05.32)
No more and no less	48 (25.53)
A little more	69 (36.70)
Much more	51 (27.13)
I encourage the child to move along to music	
Never had this habit	04 (02.13)
Much less	02 (01.06)
A little less	07 (03.72)
No more and no less	32 (17.02)
A little more	59 (31.38)
Much more	84 (44.68)
I teach the child new songs	
Never had this habit	09 (04.79)
Much less	04 (02.13)
A little less	12 (06.38)
No more and no less	46 (24.47)
A little more	55 (29.26)
Much more	62 (32.98)
The child is exposed to different types of music	
Never had this habit	02 (01.06)
Much less	07 (03.72)
A little less	10 (05.32)
No more and no less	40 (21.28)
A little more	62 (32.98)
Much more	67 (35.64)
I listen to music or play a musical instrument to socially connect with the child(ren) under my care (children, stepchildren, grandchildren, nephews)	
Never had this habit	68 (36.17)
Much less	16 (08.51)

A little less	11 (05.85)
No more and no less	0 (0)
A little more	46 (24.47)
Much more	47 (25.00)
I play a musical instrument or recorded music to change my child(ren)'s mood or physical state (energize, relax)	
Never had this habit	47 (25.00)
Much less	07 (03.72)
A little less	04 (02.13)
No more and no less	01 (00.53)
A little more	58 (30.85)
Much more	71 (37.77)

Note: Frequency (Percentage).

Table S5. Parent-reported changes in children's non-musical recreational activities. Questions focused on how often children engaged in each item during social distancing compared to a typical day before social restrictions imposed by health authorities in Brazil.

The child plays of telling stories not from a book	
Never had this habit	15 (07.98)
Much less	06 (03.19)
A little less	21 (11.17)
No more and no less	44 (23.40)
A little more	52 (27.66)
Much more	50 (26.60)
The child draws, paints, or make things	
Never had this habit	06 (03.19)
Much less	07 (03.72)
A little less	12 (06.38)
No more and no less	18 (09.57)
A little more	68 (36.17)
Much more	77 (40.96)
The child plays indoors with toys or games	
Never had this habit	03 (01.60)
Much less	05 (02.66)
A little less	09 (04.79)
No more and no less	17 (09.04)
A little more	59 (31.38)
Much more	95 (50.53)
The child plays outdoors	
Never had this habit	02 (01.06)
Much less	65 (34.57)
A little less	49 (26.06)
No more and no less	20 (10.64)
A little more	29 (15.43)
Much more	23 (12.23)
The child does physical activities	
Never had this habit	03 (01.60)
Much less	59 (31.38)
A little less	64 (34.04)
No more and no less	29 (15.43)
A little more	24 (12.77)
Much more	09 (04.79)

Note: Frequency (Percentage).
